# Designer bioemulsifiers based on combinations of different polysaccharides with the novel emulsifying esterase AXE from *Bacillus subtilis* CICC 20034

**DOI:** 10.1186/s12934-019-1221-y

**Published:** 2019-10-10

**Authors:** Weiyi Tao, Junzhang Lin, Weidong Wang, He Huang, Shuang Li

**Affiliations:** 1 College of Biotechnology and Pharmaceutical Engineering, Nanjing Tech University, Nanjing, 211816 People’s Republic of China; 2 School of Pharmaceutical Sciences, Nanjing Tech University, Nanjing, 211816 People’s Republic of China; 3 Oil Production Research Institute, Shengli Oil Field Ltd. Co. SinoPEC, Dongying, China

**Keywords:** Bioemulsifier, AXE (acetyl xylan esterase), Emulsifying activity, Polysaccharides

## Abstract

**Background:**

Bioemulsifiers are surface-active compounds, which exhibit advantages including low toxicity, higher biodegradability and biocompatibility over synthetic chemical surfactants. Despite their potential benefits, some obstacles impede the practical applications of bioemulsifiers, including low yields and high purification costs. Here, we aimed to exploit a novel protein bioemulsifier with efficient emulsifying activity and low-production cost, as well as proposed a design-bioemulsifier system that meets different requirements of industrial emulsification in the most economical way.

**Results:**

The esterase AXE was first reported for its efficient emulsifying activity and had been studied for possible application as a protein bioemulsifier. AXE showed an excellent emulsification effect with different hydrophobic substrates, especially short-chain aliphatic and benzene derivatives, as well as excellent stability under extreme conditions such as high temperature (85 °C) and acidic conditions. AXE also exhibited good stability over a range of NaCl, MgSO_4_, and CaCl_2_ concentrations from 0 to 1000 mM, and the emulsifying activity even showed a slight increase at salt concentrations over 500 mM. A design-bioemulsifier system was proposed that uses AXE in combination with a variety of polysaccharides to form efficient bioemulsifier, which enhanced the emulsifying activity and further lowered the concentration of AXE needed in the complex.

**Conclusions:**

AXE showed a great application potential as a novel bioemulsifier with excellent emulsifying ability. The AXE-based-designer bioemulsifier could be obtained in the most economical way and open broad new fields for low-cost, environmentally friendly bioemulsifiers.
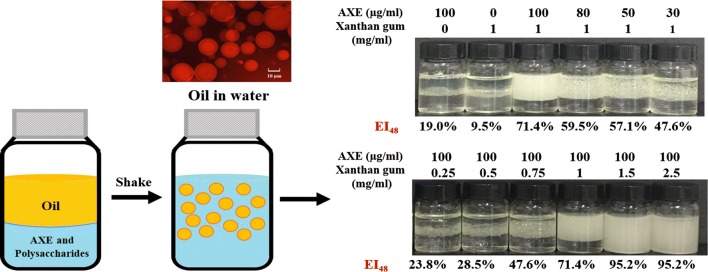

## Background

Bioemulsifiers are high-molecular-weight biopolymers produced by a wide range of diverse microorganisms, which can efficiently form and stabilize oil-in-water emulsions at a low concentration [1]. They are amphipathic complex mixtures of polysaccharides, lipopolysaccharides, lipoproteins, and proteins [1–3]. Similar to surfactants, bioemulsifiers are also capable of enhancing the bioavailability of insolubility compounds for biodegradation [4]. However, what distinguishes them from biosurfactants is that they do not significantly reduce surface and interfacial tension [1]. Notably, the ability of biosurfactants, to efficiently reduce surface tension also has adverse impacts, such as disruption and permeabilization of cell membranes [5], negative interactions with enzymes or proteins [6] and general toxicity to cells [7]. These negative can be avoided by using bioemulsifiers, which exhibit advantages including low toxicity, higher biodegradability and biocompatibility, renewable production, and even stability at extreme temperature, pH and salt concentrations [3]. These characteristics make them extraordinary valuable for industrial and environmental applications in areas such as petroleum, pharmaceutical, cosmetics, and food industries [1]. Peculiarly, bioemulsifiers were widely studied for potential usages in the petroleum industry, such as microbial enhanced oil recovery (MEOR) [8, 9] and bioremediation of contaminated soil or other environmental pollution [10]. Despite their potential advantages, some obstacles impede the practical applications of bioemulsifiers, including low yields and high purification costs. To solve these problems, many researchers have put efforts into producing and exploiting more efficient bioemulsifiers, which can be used at low concentrations.

Although the compositions of bioemulsifiers are varied and complex, studies have shown that the emulsifying activity of bioemulsifiers is highly related to their chemical compositions [11, 12]. One of the most well-known and well-studied bioemulsifier is emulsan produced by *Acinetobacter calcoaceticus* RAG-1, which is a complex of an anionic heteropolysaccharide and a protein [13, 14]. The protein in emulsan was purified and confirmed to be an esterase, which could enhance and stabilize emulsions when mixed with various polysaccharides [15–17]. Besides, another well-known bioemulsifier, alasan, composed of alanine, polysaccharides and proteins, is produced by *Acinetobacter radioresistens* KA53. There are three kinds of protein components in alasan, and they are considered as the major functional substances, among which the 45-kDa protein is demonstrated to have the highest emulsifying activity, which is even 11% higher than that of the intact alasan complex [3, 18, 19]. Two classes of proteins have been reported to have hydrophobic surface activity, hydrophobin and phasin. Hydrophobins are a group of small proteins produced by filamentous fungi which exhibit surface activity. However, the low yield of hydrophobin production, separation and purification still prevent its application as a bioemulsifiers [20]. Phasin, located on the surface of polyhydroxyalkanoates (PHA) granules, is a small amphiphilic protein which can bind to hydrophobic polymers. Applications of phasins as biosurfactant have been studied. Wei et al. [21] demonstrated that purified PhaP phasin could form stable emulsions with lubricating oil, diesel, and soybean oil. The phasin protein PhaR showed a higher emulsifying ability and could be potentially used as a biosurfactant in many areas. However, it was expressed in the inclusion body form, which brought troubles for extraction and purification [22].

We hypothesize that proteins play an indispensable role in the emulsification activity of bioemulsifiers, while polysaccharides work as stabilizers in the emulsifying systems. Based on this premise, designer bioemulsifiers can be obtained by the appropriate combination of proteins and polysaccharides components, potentially providing tailored bioemulsifier for specific hydrophobic compounds. As the protein and polysaccharide components of bioemulsifiers are easier to produce and extract on a large-scale in separate industrial processed, these combined bioemulsifiers can be provided at a relatively lower cost. However, few proteins have good emulsifying activity, studying and exploring a novel protein with potent emulsifying activity is the premise to solve the problem.

The esterase AXE from *Bacillus subtilis* CICC 20034 was previously studied by our lab, it showed high esterase activity toward cephalosporin C and 7-amino cephalosporanic acid [23], and it can also be used as a catalyst for the preparation of peracetic acid [24]. In this study, we found that AXE has an outstanding emulsifying activity and further explored its use as the protein component of designer bioemulsifiers in combination with different polysaccharides.

## Results

### Production and purification of AXE

**Fig. 1:**
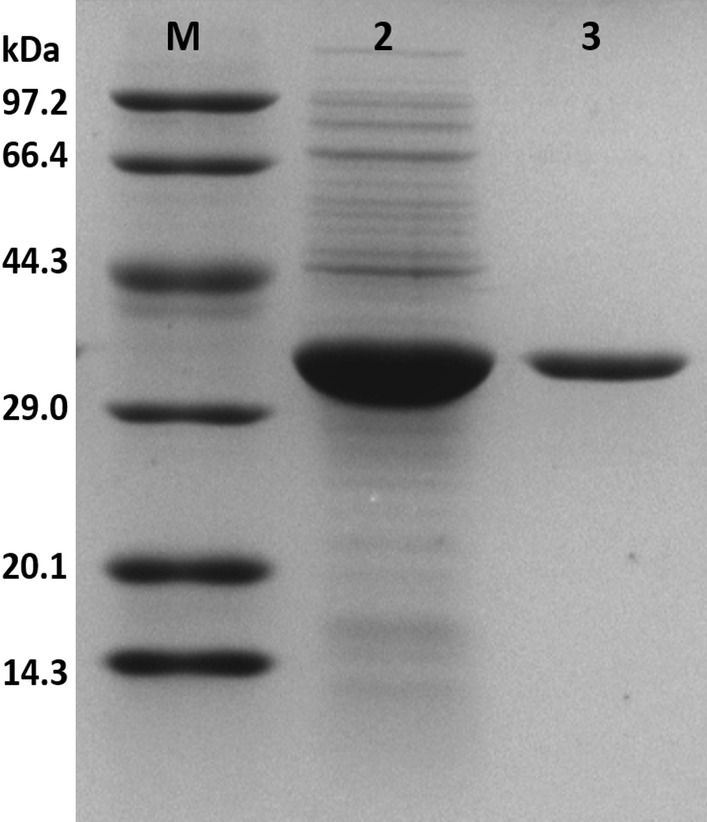
The SDS-PAGE analysis of the purified AXE. M: molecular mass markers; lane 2: crude AXE; lane 3: purified AXE

After 16 h of incubation in autoinduction medium, AXE was expressed in recombinant *E. coli* strain BL21-pET28a-Cah in soluble-form. A total of 3.209 g/L of crude AXE (AXE protein) was produced, which accounted for 72.4% of the total soluble protein. The purification was achieved using classical Ni–NTA affinity chromatography. As shown in Fig. 1, the AXE protein appeared as a single band, which was consistent with the molecular mass of 35.6 kDa. A final yield of 1.13 g/L purified AXE esterase was obtained, corresponding to a recovery rate of 35.2%.

### Study of emulsification by AXE

**Fig. 2:**
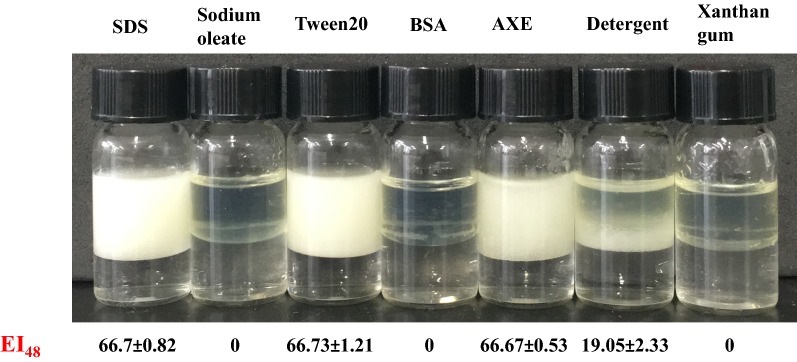
A comparison of the emulsion index of purified AXE and widely used surfactants. For comparison, some commonly used surfactants were also used, including SDS, sodium oleate, Tween 20, detergent and xanthan gum. BSA was employed as control. All the proteins and surfactants were added at a concentration of 500 µg/ml dissolved in 50 mM Tris–HCl buffer, pH 7.4

Synthetic surfactants are widely used in the industry. In this study, AXE was compared with commonly used surfactants. An aliquot comprising 2 ml of each aqueous surfactant solution (500 µg/ml in 50 mM Tris–HCl buffer, pH 7.4) and 2 ml of diesel oil were mixed and vortexed for 60 s, respectively. The emulsification values were measured 2 days later. As shown in Fig. 2, the emulsion index of AXE was similar to those of SDS and Tween 20, and much higher than those of sodium oleate, commercial detergent and xanthan gum, which indicated that AXE had good emulsifying activity.

### AXE is a bioemulsifier rather than a biosurfactant

Both biosurfactants and bioemulsifiers can form stable emulsions, but only biosurfactants have an excellent ability to reduce surface and interfacial tension, which stands in distinct contrast to bioemulsifiers [1]. Here, the interfacial tensions between diesel oil and an aqueous solution containing different surfactants or AXE were measured by the du Nouy ring method at 25 °C. The results are listed in Table 1. As expected, surfactants such as SDS and Tween 20 significantly reduced the diesel/aqueous interfacial tensions from 21.25 mN/m to 1.72 mN/m and 3.89 mN/m respectively, while biopolymers, like xanthan gum, could not reduce the interfacial tension. The interfacial tension of the solution comprising 500 µg/ml AXE was 16.09 mN/m, which was very close to the 17.41 mN/m obtained for the 500 µg/ml BAS (bovine serum albumin) solution. These results suggested that AXE was not a surface-active protein.

**Table 1:** Oil/water interfacial tension between diesel oil and solutions containing different surfactants

Oil/water phase	σ (mN/m)
Diesel/H_2_O	26.64 ± 0.02
Diesel/Tris–HCl buffer	21.25 ± 0.07
Diesel/SDS	1.72 ± 0.01
Diesel/BSA	17.41 ± 0.09
Diesel/AXE	16.09 ± 0.07
Diesel/xanthan gum	25.20 ± 0.11
Diesel/Tween 20	3.89 ± 0.02

The oil/water interfacial tension was measured at 25 °C by the du Nouy ring method, and 500 µg/ml protein or surfactant were dissolved in Tris–HCl buffer solution (50 mM, pH 7.4)

**Fig. 3:**
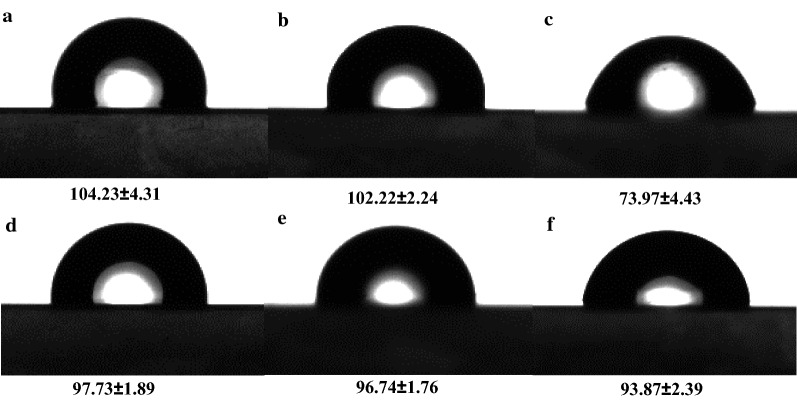
Comparison of contact angles of different surfactants dropped on the biaxially oriented polypropylene (BOPP) films. **a** Water, **b** 500 µg/ml BSA, **c** 500 µg/ml SDS, **d** 500 µg/ml AXE, **e** 1000 µg/ml AXE, **f** 2000 µg/ml AXE

To further study the surface activity of AXE, we compared the water contact angles of drops of AXE solution with those containing different surfactants on the hydrophobic surface (BOPP film). Samples comprising 1 μl of water, BSA (500 μg/ml), SDS (500 μg/ml), AXE (500 μg/ml), AXE (1000 μg/ml) and AXE (2000 μg/ml) were respectively dropped on BOPP films. As shown in Fig. 3, the contact angles of water and BSA were 104° and 102°, respectively. SDS produced the smallest contact angles of 74°. The contact angle was reduced from 97° to 93° when the AXE concentration increased from 500 to 2000 μg/ml. This result was consistent with the measurement of oil–water interfacial tension, which indicated that AXE had a weaker ability to reduce surface tension.

### Effects of pH, salt concentration, and temperature on the stability of emulsions generated using AXE

**Fig. 4:**
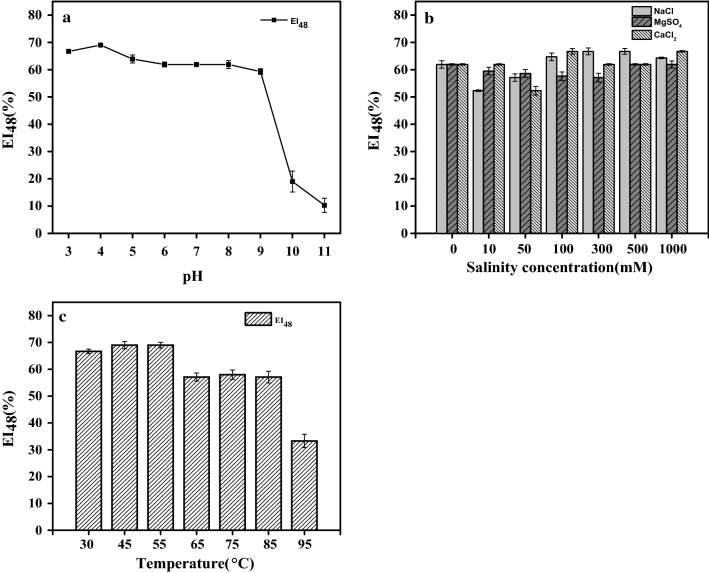
Effect of pH, salt concentration and temperature on the emulsifying activity of AXE. Diesel was used as the model hydrophobic substrate, and the concentration of AXE was 500 μg/ml in 50 mM Tris–HCl buffer, pH 7.4

In practical application, pH, salt concentration and temperature are essential environmental factors that must be considered when assessing the activity and stability of emulsifiers. The impact of pH on the emulsifying activity of AXE was measured at the range of pH 3–11 (Fig. 4a). The AXE emulsion was stable at pH values under 9. AXE showed higher emulsifying activity at pH 3–4, and the activity significantly decreased at alkaline conditions over pH 10. Several studies have reported that pH greatly affected the stability of surfactants and emulsifiers. However, the alkaline condition was mostly reported to have a positive effect on the stability of biosurfactants and increased the emulsifying activity. Alasan and the 45 kDa protein from *Acinetobacter radioresistens* KA53 were both active at pH from 8 to 9 [3]. The emulsifying activity of surfactin produced from *B. subtilis* ATCC 21332 was much better at pH 11 than under acidic conditions, and the activity was almost completely lost at pH values below 4.0 [25]. By contrast, AXE favoured acidic conditions and almost completely lost the ability to form emulsions at pH 11. As showed in Fig. 4b, AXE emulsion exhibited excellent stability over a range of NaCl, MgSO_4_ and CaCl_2_ concentration from 0 to 1000 mM. There was even a slight increase in emulsifying activity at salt concentration over 500 mM. This result demonstrated that inorganic salts had no apparent effect on the emulsifying activity of AXE, which could be an advantage in the practical application. As shown in Fig. 4c, AXE retained most of its emulsifying activity after heating for 1 h at temperatures from 30 to 55 °C, while a slight decrease was observed above 65 °C. The emulsion layer started to collapse, and only approximately half of the original emulsifying activity remained after heating at 95 °C.

### Emulsification of different hydrophobic substrates

Most bioemulsifiers and microbial surfactants preferentially emulsify a subset of hydrophobic substrates, which is essential for their usage in industrial processes. The emulsifying activities of AXE toward various hydrophobic substrates were shown in Table 2. At an AXE concentration of 500 μg/ml, benzene, xylene and diesel were the most well-emulsified substrates, with an emulsion ratio of approximately 66.7%. *n*-Hexane and *n*-octane were also suitable substrates for emulsification. In this study, AXE was active with various hydrophobic substrates, and a stable emulsion was formed in many different systems when the AXE concentration was higher than 200 μg/ml.

**Table 2:** Emulsifying activity (EI_48_) toward various hydrophobic substrates with AXE at different concentrations

Oil/hydrophobic	AXE concentration (µg/ml)			
	500	200	100	50
*n*-Hexane	61.90 ± 0.56	48.10 ± 3.02	28.57 ± 2.39	14.29 ± 2.56
*n*-Hexadecane	9.52 ± 1.28	4.77 ± 0.78	4.77 ± 0.56	0
*n*-Octane	61.90 ± 0.92	52.38 ± 0.56	23.81 ± 2.37	19.05 ± 1.47
Benzene	66.67 ± 1.21	66.67 ± 0.77	66.13 ± 1.66	42.33 ± 1.84
Xylene	66.67 ± 0.81	66.67 ± 1.21	54.76 ± 1.66	28.57 ± 0.91
Kerosene	47.62 ± 1.84	47.62 ± 1.59	27.62 ± 1.07	23.81 ± 1.33
Diesel	66.67 ± 0.93	61.90 ± 1.77	23.81 ± 2.82	0
Soybean oil	23.81 ± 1.73	23.81 ± 2.54	14.29 ± 1.85	0
Lubricating oil	19.05 ± 2.98	4.76 ± 0.77	4.76 ± 1.98	4.76 ± 1.33
Olive oil	31.90 ± 1.72	27.12 ± 2.04	27.67 ± 1.49	22.83 ± 2.26

The reaction was performed in identical cylindrical glass vials with a reaction volume of 2 ml purified AXE solution in 50 mM Tris–HCl buffer, pH 7.4, and 2 ml oil or hydrophobic substrates after vortexing. All samples were stored for 2 days before observation

The results represent the mean ± SD over at least three replicates

### Hydrophobic regions near the C-terminus of AXE

We tried to compare the sequences of RAG-1 esterase and AXE to further explore the mechanism underlying its emulsifying activity. However, there is only 10.4% identity between AXE and RAG-1 esterase. Bach et al. have proved that the C-terminal 100 amino acids are as active as the whole RAG-1 esterase in enhancing emulsification, and it is necessary for emulsification enhancement as well [16]. Considering the importance of the C-terminal amino acids, we further compared the C-terminus hydrophobic regions of the AXE with RAG-1 esterase. The results were shown in Table 3. The grand average of hydropathy (GRAVY) value was calculated using the ProtParam tool. Neither AXE nor RAG-1 esterase was hydrophobic proteins, and their average hydrophobic values of sequence regions from 202 to 308 were − 0.236 and − 0.416, respectively. There were six hydrophobic regions in the C-terminus of RAG-1 esterase, whereas only four in AXE. However, the hydrophobic value of the four regions in AXE was higher than RAG-1 esterase’s, which means that the four regions were more hydrophobic. Four hydrophobic regions in 45-kDa alasan protein were also proved to be necessary for its emulsifying activity, as well as many studies had shown that hydrophobic regions on the surface of protein might be related to the alignment of the protein at interfaces, which further affected its emulsifying properties [26–28]. Thus, we considered that the four hydrophobic regions were highly correlated to the AXE emulsifying activity.

**Table 3:** Hydrophobic regions of the AXE compared with homologous regions of RGA-1 esterase

Sequence position	AXE		RAG-1 esterase	
	Sequences	GRAVY^a^	Sequences	GRAVY^a^
223**–**229	LEINSFF	0.871	VSPLFDD	0.200
236**–**240	ETEKT	−2.460	TLVQV	1.600
245**–**250	LAYFDI	1.350	ILLDDS	0.717
264**–**269	SIGLID	1.350	VHFKLY	0.400
276**–**281	TVFAAY	1.433	FQMFNA	0.383
289**–**293	QLKVY	−0.140	ALADI	1.680

^a^The GRAVY value for a peptide was calculated using the ProtParam tool. It is the sum of hydropathy values of all the amino acids, divided by the number of residues in the sequence [29]

### Designed emulsifier based on polysaccharide–AXE mixtures

As emulsions are thermodynamically unstable systems that tend to break down over time, polysaccharides are widely used to stabilize the oil-in-water emulsion. The high-molecular-mass bioemulsiers produced by microbes are amphipathic polysaccharides, proteins, lipopolysaccharides, lipoproteins or complex mixtures of these biopolymers [1]. The natural polysaccharide-protein conjugates such as RAG-1 emulsan and alasan exhibit efficient emulsification activity and have been used in industrial applications, which inspired us to design novel bioemulsifiers comprising mixtures of AXE and a wide variety of polysaccharides. The results are shown in Table 4. In addition to methylcellulose, all the polysaccharides listed in Table 4 can be mixed with AXE to form new bioemulsifers with better emulsifying activities (all polysaccharides have no emulsifying activity themselves at 1 mg/ml, except for xanthan gum, EI_48_ was 9.5). Among them, the xanthan gum–AXE mixture has the highest emulsification Index (76.73), which is 20% higher than that of AXE alone. Compounds with gum arabic and welan gum also increase the emulsifying activity, around 12% and 13% respectively. Since the major force preventing oil droplet coalescence in protein emulsion is electrostatic repulsion [30], it is necessary to measure the zeta potential to evaluate the effect of polysaccharides on oil droplets condensing in AXE emulsions, and the results are shown in Table 4. All polysaccharide–AXE mixtures exhibit a negatively charged zeta potential, and the zeta potential of 200 µg/ml AXE is − 22.3 mV. The xanthan gum and welan gum are both anionic polysaccharides and contribute to the significant decrease in the zeta potential, − 49.3 mV and − 42.9 mV respectively. As the higher absolute value of zeta potential is, the more stable emulsion is, therefore, xanthan gum and welan gum improve the stability of the emulsion by increasing the electrostatic repulsion between AXE covered oil droplet.

**Table 4:** Emulsification activity (EI_48_) and zeta potential of mixtures

Polysaccharides (1 mg/ml)	Emulsification index (EI_48_) (200 µg/ml AXE)	Zeta potential (mV)
AXE	61.90 ± 0.35	− 22.3 ± 1.4
Agarose	66.67 ± 1.20	− 24.5 ± 0.7
Chitosan	66.72 ± 0.83	− 22.6 ± 1.8
Starch	67.04 ± 1.38	− 17.8 ± 1.0
Dextrin	66.67 ± 0.22	− 24.6 ± 1.2
Gum arabic	69.85 ± 2.10	− 34.2 ± 2.2
Xanthan gum	76.73 ± 2.18	− 49.3 ± 1.8
Gelatin	61.90 ± 1.43	− 26.2 ± 2.6
Methylcellulose	47.60 ± 3.32	− 11.3 ± 0.8
Welan gum	70.33 ± 1.22	− 42.9 ± 2.5

**Fig. 5:**
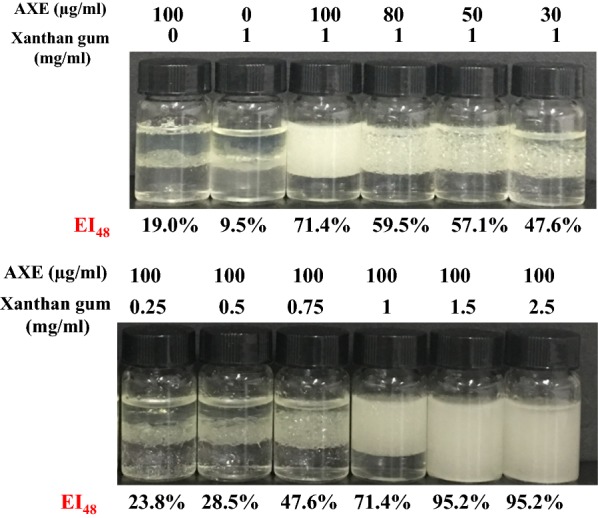
Emulsifying activity (EI_48_) of mixtures comprising different concentration of xanthan gum and AXE

Since xanthan gum is an anionic polysaccharide produced by the bacterium *Xanthomonas campestris*, its unusual thickening properties and rheology make xanthan gum an excellent emulsion stabilizer [31], and it has been one of the most commonly employed polysaccharides for industrial emulsification purposes. Therefore, combined in a single system under appropriate conditions (concentrations, protein-to-polysaccharide ratio), the properties of AXE and xanthan gum would be of great interest in improving emulsion stability. Hence, we further investigated the xanthan gum–AXE mixture and adjusted its composition. As shown in Fig. 5, the emulsifying activity of xanthan gum–AXE combination was much higher than either xanthan gum or AXE alone. When the content of xanthan gum was 1 mg/ml, the xanthan gum–AXE mixture could form a stable emulsion (EI_48_ 71.4%) even after reducing the concentration of AXE to 100 µg/ml. Moreover, the emulsifying activity of the mixture remained at 47.6, even when the concentration of AXE was decreased to 30 µg/ml. At an AXE content of 100 µg/ml, increasing xanthan gum content to 1.5 mg/ml yielded a mixture that almost fully emulsified the diesel oil/water mixture. Besides, a more stable emulsion layer can be observed in xanthan gum–AXE mixture. Even after 14 days, the EI of the xanthan gum–AXE mixture (1 mg/ml xanthan gum, 100 µg/ml AXE) remained unchanged, about 70.2%. Thus, the emulsions formed by xanthan gum–AXE mixtures were more stable than AXE alone, and the results were consistent with the zeta potential results.

## Discussion

Many studies have shown that protein, as one of the essential components in bioemulsifiers, plays a vital role in enhancing stability and improving emulsification properties, while few reports that protein, itself can be acted as an emulsifier to form a stable emulsion with hydrophobic solvent. This is the first detailed investigation of the esterase AXE from *Bacillus subtilis* CICC 20034, which exhibits excellent emulsifying activity comparable to commercial chemical emulsifiers.

AXE could form stable emulsions with different hydrophobic substrates, and showed better emulsifying activity on benzene, xylene and diesel, while the AXE emulsion index of kerosene was lower than that of diesel. Considering that diesel is a mixture of aromatic and aliphatic hydrocarbons in the size range of C4-C12, while kerosene consists of about ten different hydrocarbons with sized of C16-C20, the results indicated that AXE was less suitable for long aliphatic hydrocarbons. Additionally, AXE had low emulsifying activity on soybean oil and lubricating oil. As expected, the stability of the emulsions increased with ascending AXE concentration. This phenomenon could also be observed when phasin protein used as an emulsifier [21]. The emulsification preference of different hydrophobic substrates by protein bioemulsifiers was summarized in Table 5. In contrast to alasan and the 45-kDa protein Alna from *Acinetobacter radioresistens* KA53 [3], as well as emulsan from *Acinetobacter calcoaceticus* RAG-1, AXE was able to efficiently emulsify short-chain aliphatic and aromatic hydrocarbons, such as *n*-hexane, benzene and xylene. AXE had better emulsification activity for short-chain substrates than PS-bioemulsifier from *Pseudomonas stutzeri* [32]. In addition, although AXE had a lower emulsifying activity on soybean oil and lubricating oil than the phasin proteins PhaP and PhaR, there was no apparent difference between their emulsifying activities toward diesel [21, 22]. Thus, AXE appeared to stand out for its ability to efficiently emulsify short-chain aliphatic and aromatic substrates. AXE also showed excellent stability under extreme conditions such as high temperature (85 °C), acidic pH, and high salt concentrations.

**Table 5:** Preference for different hydrophobic substrates of various bioemulsifiers

Emulsifier	Component	Molecular weight	Hydrophobic substrate specificity					
			Diesel	Soybean oil	Lubricating oil	Aliphatic (< C8)	Aliphatic (C10 < C18)	Aromatic
AXE	Protein	35.6 kDa	+++	++	++	+++	+	+++
PhaP	Protein	14.6 kDa	+++	+++	+++	NA	NA	NA
PhaR	Protein	25 kDa	+++	+++	+++	NA	NA	NA
RAG-1 Emulsan	Polysaccharide-protein	980 kDa	+++	NA	NA	–	–	–
Alasan	Polysaccharide-protein	1 MDa	NA	+	NA	+	+++	+
AlnA	Protein	35.7 kDa	NA	NA	NA	+	++	NA
Ps-bioemulsifier	Polysaccharide-protein	NA	+++	NA	NA	+	++	+++

NA: Data not available;

+++: substrate was highly emulsified;

++: substrate was slightly emulsified;

+: substrate was poorly emulsified;

–: substrate was not emulsified

It is worth noting that AXE is a bioemulsifier rather than a biosurfactant. Phasin protein PhaP can efficiently reduce the oil–water interfacial tension and water surface tension (below 50 mN/m) [28]. Similarly, the 45-kDa protein from alasan (AlnA) can reduce the water surface tension from 70.7 to 37.89 mN/m [4]. In addition, PhaP and PhaR are even more capable of reducing the water contact angle than SDS. By contrast, AXE cannot significantly reduce the surface tension. The AXE solution almost has the same surface tension as the Tris–HCl buffer (53.74 mN/m). Thus, the poor ability of AXE to decrease water contact angle and interfacial tension distinguish it from biosurfactant. To our knowledge, AXE is the only known protein that can efficiently emulsify diesel but is less effective at reducing interfacial tension. Thus, it may offer distinct advantages over surfactants, such as lower toxicity and higher biocompatibility.

Many studies have shown that the emulsifying ability of proteins is related to their tertiary structure [21]. The hydrophobic regions of some proteins can tightly bind to the oil–water interfaces or hydrophobic substrates [28]. The hydrophobic regions on the surface of alasan are considered to be responsible for its surfactant properties [18]. Furthermore, molecular modelling of the structure of PhaP revealed that it contains multiple amphipathic helices forming coiled coils, which are essential to its surfactant properties [27, 28, 33]. Thus, it is crucial to analyze the structure of AXE to explain its emulsifying activity. However, AXE has a very low homology with the 45 kDa protein from alasan (the protein with the highest emulsifying activity), only 13.5%. Similarly, the identity between AXE and RAG-1 esterase is only 10.4%. Further compared the C-terminus hydrophobic regions of the AXE with RAG-1 esterase, we found that neither AXE nor RAG-1 esterase is hydrophobic proteins. There are four hydrophobic regions in AXE, and the hydrophobic values of the four regions in AXE were higher than those of the RAG-1 esterase, which may explain its interaction with hydrocarbon.

In our previous work, we have successfully prepared AXE esterase from a low-cost autoinduction medium, and the AXE was expressed in soluble-form and its protein expression account for 72.4% of the total soluble protein. However, the esterase from RAG-1 emulsan was overexpressed in *Escherichia coli* BL21(DE3) [34], among which the most of the recombinant esterase was expressed in inclusion bodies (80–90%). Similarly, PhaP protein was also expressed in the inclusion body form [22]. The necessary renaturation of the recombinant esterase from inclusion bodies would increase the cost and complexity of the overall process. By contrast, the esterase AXE could be produced in a soluble form. This enables easy production and purification, which offers a very economical potential protein for use as bioemulsifiers.

We also proposed a designed-bioemulsifier system that can be used to develop novel bioemulsifiers by combining AXE with a wide variety of polysaccharides, which enhanced the emulsifying activity and further lowered the concentration of AXE needed in the mixture. The results suggest that AXE protein and polysaccharides both partake in creating novel emulsions with improved stability and functionality. By adjusting the concentration of the polysaccharides and AXE, the novel designer bioemulsifier, which meets the requirements of industrial emulsification, can be obtained in the most economical way. For example, in xanthan gum–AXE system, the required concentration of AXE was drastically reduced, which in turns decreased the cost in practical applications. The designed bioemulsifier system also meets the needs of bioemulsifier application in different fields by selecting various polysaccharides, like starch. These designed bioemulsifiers can, therefore, be potentially used in many different areas, including the food, pharmaceutical and cosmetics industries. To our best knowledge, other systems such as the proteins from *Acinetobacter calcoaceticus* BD4, BD413 and the RAG-1 emulsan cannot form stable emulsions without polysaccharides [12, 17].

## Conclusions

This is the first detailed investigation of the esterase AXE from *Bacillus subtilis* CICC 20034, which exhibits excellent emulsifying activity comparable to commercial chemical emulsifiers and can form stable emulsions with different hydrophobic substrates even at low pH, high salt concentrations and high temperatures. We also propose a designed-bioemulsifier system that can be used to develop novel bioemulsifiers by combining AXE with a wide variety of polysaccharides, which enhanced the emulsifying activity and further lowered the concentration of AXE needed in the mixture. This study thus opens broad new fields of application for AXE-based, low-cost, environmentally friendly bioemulsifiers.

## Methods

### Production and purification of AXE protein

The AXE encoding gene *Cah* was amplified from *Bacillus subtilis* CICC 20034 and the recombinant *E. coli* strain BL21-pET28a-Cah was constructed in our lab [24]. The crude AXE esterase could be efficiently produced in a low-cost autoinduction medium in a 5-L jar fermenter at an agitation rate of 200 rpm and aeration of 1.5 v/v/m. The autoinduction medium contained 15 g/L peptone (Angel Yeast Co., Ltd, China), 25 g/L yeast extract (Angel Yeast Co., Ltd, China), 10 g/L NaCl, 0.3 ml/L glycerin, 2 g/L glucose, and 2 g/L lactose. After incubation at 30 °C for 16 h, the cells were harvested by centrifugation at 10,000×*g* for 10 min, resuspended in 100 mM Tris–HCl buffer (pH 7.4) and disrupted using a high-pressure homogenizer. The crude lysate was cleared by centrifugation (10,000×*g* for 30 min at 4 °C), and the supernatant was loaded onto a Ni–NTA affinity chromatography column (Ni–NTA HisTrap FF CRUDE 5 × 5 ML, GE Healthcare, USA) pre-equilibrated with washing buffer (50 mM Tris–HCl pH 7.4, 500 mM NaCl). The protein was washed and eluted using imidazole washing buffer (50 mM Tris–HCl pH 7.4, 500 mM NaCl, 10 mM imidazole) and elution buffer (50 mM Tris–HCl pH 7.4, 500 mM NaCl, 500 mM Imidazole), respectively. At the end of the procedure, the imidazole in the purified solution was removed by dialysis. The purified AXE enzyme was concentrated using a 10 kDa Ultrafiltration cartridge (Amicon Ultra-15, Merk Millipore, Germany) and preserved in 50 mM Tris–HCl buffer, pH 7.4, at 4 °C. The purified AXE was analyzed by SDS-PAGE, and the concentration was determined using the Bradford method with bovine serum albumin as the standard.

### Preparation of emulsions

The emulsification index of AXE was determined using diesel as the substrate and compared with that of other commonly used surfactants and proteins, including sodium dodecyl sulfate (SDS; Aladdin, China), sodium oleate (3A Chemical Technology Co., Ltd, China), Tween 20 (3A Chemical Technology Co., Ltd, China), bovine serum albumin (BSA; Sigma-Aldrich, USA), detergent (Liby, China) and xanthan gum (USP, Aladdin, China). An aliquot comprising 2 ml of each aqueous surfactant solution (500 µg/ml protein or chemical surfactant dissolved in 50 mM Tris–HCl buffer, pH 7.4) and 2 ml of diesel were mixed, respectively. The mixtures of oil and aqueous solution were then treated for 60 s. All samples were stored in a dark room at 25 °C and emulsification values were measured after two days.

The emulsification index (EI_48_) was calculated according to the following formula:

$$ {\text{Emulsification index }}\left( {{\text{EI}}_{48} } \right) = \frac{\text{Height of the emulsion layer}}{\text{Height of all layers}} \times 100\% $$

The assay was performed in identical cylindrical glass vials, and three parallel samples were measured for every emulsification value.

### Stability of AXE at a range of pH values, temperatures, and salt concentrations

A 500 μg/ml solution of purified AXE in 50 mM Tris–HCl buffer, pH 7.4, was used to analyze the effects of temperature, salinity and pH values on its emulsifying activity with diesel oil. For the thermal stability study, the AXE solution was incubated for 1 h in a water bath at temperatures from 30 to 95 °C. The salinity stability was tested with NaCl, MgSO_4_, and CaCl_2_ at concentrations from 10 to 1000 mM. For determining pH stability, the AXE solution was adjusted to different pH values (3–11) with dilute HCl or NaOH solutions.

### Measurement of oil/water interfacial tension

The interfacial tension at the oil/water interface was measured at 25 °C via the du Nouy ring method [35], using a QJZY-1 automatic surface tensiometer (Shanghai Pingxuan Co., Ltd, China). Each sample was measured at least three times.

### Water contact angle measurements

Biaxially oriented polypropylene (BOPP) film (Thickness 0.01 mm, Sigma-Aldrich, USA) was immobilized on a glass slide for the water contact angle measurements. Aliquots comprising 1 μl volume of various surfactant solutions were dropped on BOPP film, and the drops were imaged using an optical contact angle meter (Dropmeter A-100P, MAIST Vision I Co. Ltd., China). The experiments were carried out at room temperature (25 °C), and each sample was tested three times.

### Emulsifying activity toward various hydrophobic substrates

The Emulsifying abilities of AXE were examined using multiple hydrophobic substrates, such as *n*-hexane, hexadecane, octane, xylene (3A Chemical Technology Co., Ltd, China), kerosene, diesel (China National Petroleum Co. Ltd), soybean oil (Yihai Kerry food marketing co. LTD, China), lubricating oil (Mobil, USA), and olive oil (Yihai Kerry food marketing co. LTD, China). An aliquot comprising 2 ml of purified AXE in 50 mM Tris–HCl pH 7.4 at various protein concentrations and 2 ml of the hydrophobic substrate were mixed, respectively. The emulsifying activity was assessed via the emulsification index (EI_48_) described above.

### Designed mixed polysaccharide–AXE bioemulsifiers

The experiment was first carried out using diesel as the substrate and 2 ml of a mixture of an aqueous solution comprising 200 µg/ml AXE and 1 mg/ml various polysaccharides dissolved in 50 mM Tris–HCl buffer, pH 7.4. The emulsification values were measured after 2 days. The amounts of polysaccharides and AXE protein were further optimized after comparing emulsification values of various mixed systems.

### Measurement of the zeta potential

The zeta potential analyses were performed with Zetasizer Nano ZS90 (Malvern Instruments). Each sample was dispersed in 50 mM Tris–HCl buffer, pH 7.4, and loaded into the capillary cell. Each measurement was repeated three times.

## Data Availability

Not applicable.
